# Demand for fish in Great Britain is driven by household income and taste

**DOI:** 10.1017/S1368980025000217

**Published:** 2025-03-19

**Authors:** Shashika D Rathnayaka, Cesar Revoredo-Giha, Baukje de Roos

**Affiliations:** 1 The Rowett Institute, University of Aberdeen, Aberdeen AB25 2ZD, UK; 2 Food Marketing Research Team, Scotland’s Rural College (SRUC), Edinburgh EH9 3JG, UK

**Keywords:** Fish, Household groups, Rotterdam model, Demand elasticities, Taste

## Abstract

**Objective:**

Fish is high in nutrients that provide a range of health benefits, but people in Great Britain only consume around half the amount that is recommended. This study analysed the demand for fish for consumption at home across different household groups in Great Britain.

**Design:**

Using a Rotterdam demand model, price and income elasticities were estimated for eleven fish groups across seven household groups. To investigate changes in fish demand, we decomposed growth in fish demand into income, relative price and change in taste and seasonality.

**Setting:**

The data used for our analysis were drawn from the Kantar Worldpanel dataset for Great Britain for the period from 2013 to 2021.

**Participants:**

12 492 households in Great Britain.

**Results:**

Families with children consistently allocated a lower share of their grocery spending on fish and preferred to purchase ready-to-use and convenient fish products, compared with households without children. Purchases of chilled fresh/smoked oily fish, canned oily fish and frozen processed fish led spending across demographic groups, whilst purchases of canned oily fish showed minimal growth. The demand for most fish products across household groups was price inelastic, indicating that the percentage change in the quantity of fish demanded increased or fell by less than the percentage change in price.

**Conclusions:**

Income and taste were identified as significant determinants of demand changes across all household groups, while price only played a prominent role in demand changes for certain fish groups. Thus, increasing fish consumption, especially in lower-income groups, who do not usually consume much fish, may require a different intervention than simply making fish more affordable.

Fish is important for human health – fish consumption is associated with a decreased risk of CVD and dementia^(1–3)^, and nutrients in fish, including *n*-3 fatty acids, play an important role in cognitive development and immune regulation^(4,5)^. Fish is also a valuable contributor to the reference nutrient intakes for a range of micronutrients, including vitamins D and B_12_, Fe, Se, Zn and Ca, and therefore, fish consumption may contribute to alleviating prevalent micronutrient deficiencies, especially in developing countries^(6,7)^. Furthermore, fish is considered an important element of more sustainable diets^(8)^. Greenhouse gas emissions linked to fish consumption are significantly lower than those linked to consumption of red meat and pork^(9,10)^.

Despite the country being a significant fish producer and having food-based dietary guidelines for fish, consumption in the UK is low and has not changed in the past decades^(11)^. Per-person weekly seafood consumption in the UK in 2019, at home and away from home, was 152·8 g, marking a 3·9 percent decrease compared with 2 years prior^(12)^, and translating to just over one portion of fish, or 140 g, per person per week. This decline in UK seafood purchases has been primarily attributed to a 25 percent reduction in retail purchases over the past decade, resulting in approximately $7·7 billion lost in retail seafood sales^(12)^. Previous demand analysis in the UK suggested that the quantity of fish demanded by consumers determines retail prices rather than the other way around^(13)^. Also, retail demand for fourteen relevant UK fish products, measured between 1992 and 2001, indicated that haddock, salmon, flatfish, shellfish and smoked fish were expenditure elastic, whilst most of the fourteen species were own-price inelastic, indicating that the percentage change in quantity demanded increases or falls by less than the percentage change in price. This means that demand for these five fish species is decreasing if household expenditure or household income is decreasing, whilst price changes have less of an impact on demand^(14)^. Another study^(15)^ found that all canned tuna products had negative and inelastic price elasticities, again suggesting that demand remains relatively unaffected by price changes.

In a departure from the previous studies, we set out to compare fish demand patterns across different household groups in Great Britain, as household structure plays a significant role in shaping dietary choices. Studies in high-income countries consistently show how household composition, including factors like the presence of children and the life stage of family members, influences food consumption, reflecting diverse family needs and preferences. Moreover, variations in resources (household budget and time) and food expenditure relative to household composition affect food availability at home^(16–18)^. We also set out to decompose changes in consumer demand for fish from 2013 to 2021 into income, relative price and change in taste and seasonality. This would allow us to identify specific drivers of demand changes for different fish product groups since changes in demand frequently arise from simultaneous shifts in commodity prices, total expenditure, tastes and seasonal influences^(19–22)^. Analysing the responses of fish demand to changes in prices and income is paramount when assessing how technological advancements, infrastructure development or economic policies could shape the future landscape of fish production, consumption and trade across diverse fisheries and aquaculture products. Furthermore, understanding fish demand across various fish product categories can highlight which are most promising for capturing expanded market shares or for interventions that could enhance fish consumption and therefore public health outcomes.

## Methodology

### Data

The data used for our analysis were drawn from the Kantar Worldpanel dataset, containing weekly acquisition data of food and drink purchases for consumption at home for 12 492 Great Britain households, covering the period January 2013 to December 2021 (the dataset does not cover Northern Ireland). Participating households were asked to record all purchases using barcode scanners and send digital images of cash register receipts. The till receipts were used to provide information on prices and place of purchase. The Kantar dataset contains information on purchases, including price, quantity purchased, the supermarket from which the product was purchased and the type of promotion used.

**Figure 1.**
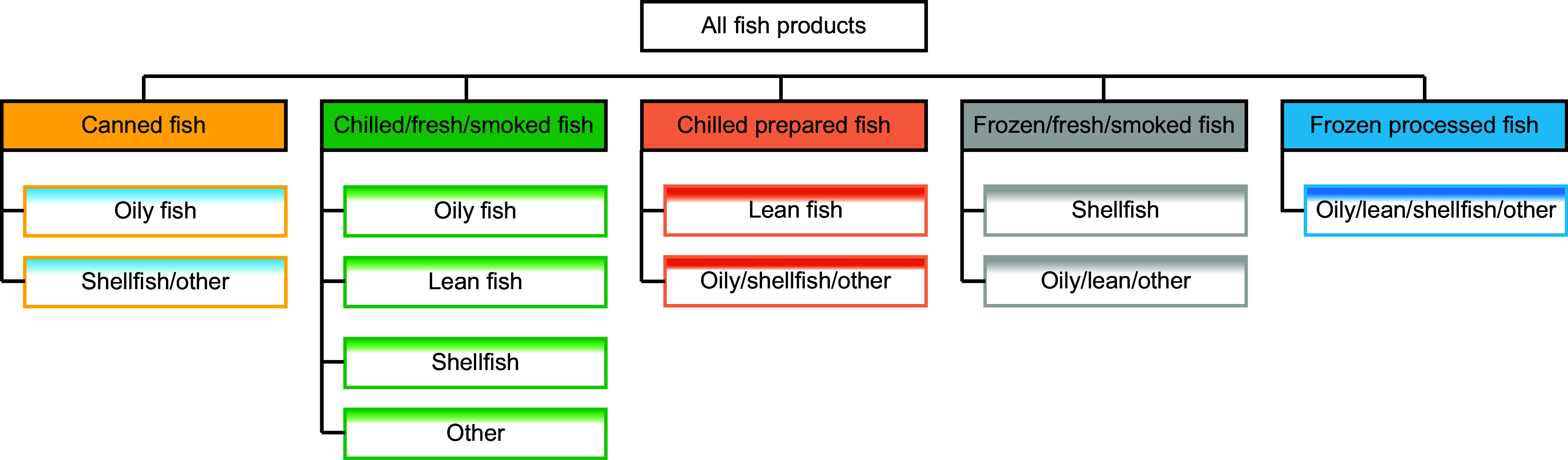
Categorisation of fish products into five main and twenty subgroups. Subgroups were aggregated to accommodate zero consumption levels, resulting in eleven distinct fish subgroups.

For our time series analysis, we aggregated the weekly data into ‘statistical months’, each comprising 4-week periods, resulting in 13 periods per year, or 117 total observations over the study period. This aggregation was conducted at the population level for each of the seven Kantar-predefined household groups^(23)^, allowing us to examine aggregate consumption and expenditure patterns for each group over time. We systematically categorised all fish products into five main categories: canned fish, chilled or fresh smoked fish, chilled prepared fish, frozen fresh or smoked and frozen processed. Each category was further subdivided into four distinct groups: oily fish products, lean fish products, shellfish products and other fish products, resulting in a total of twenty fish subgroups. Subgroups were aggregated to accommodate zero consumption levels, ultimately arriving at eleven distinct fish and seafood groups (Figure 1). Moreover, based on classification by Kantar, seven household groups were considered: pre-family (< 45 years, no children), young family (any age, children aged 0–4 years), middle family (any age, children aged 5–9 years), older family (any age, children aged 10+), older dependents (age 45+ years, no children, 3+ adults), empty nest (age 45–65 years, no children, 1–2 adults) and retired (age 65+ years, no children, 1–2 adults). The basis for this classification was the age of the household wife and the number of adults and children in the family.

### Model specification

In this study, we opted for the Rotterdam demand model^(24,25)^ because it aligns with demand theory^(26)^, exhibits excellent aggregate properties^(27)^, can be interpreted as approximations to the true unknown ones^(28)^ and is characterised by simplicity, making it easy to estimate and interpret parameter values. This model also permits the incorporation of external factors influencing demand, either with or without imposing theoretical constraints^(29)^. Given the presence of autocorrelation in time series data, employing a differential model is advantageous for mitigating this issue and enhancing the robustness of parameter estimation and interpretation.

Considering the basic specification of the Rotterdam demand model, *i* ^th^ equation of our estimated model is given by
(1)

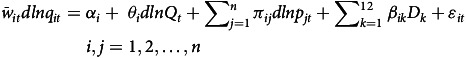




In equation (1) 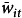 is the arithmetic average of the budget shares in period *t* and *t*-1, 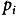 and 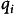 , are the price and the quantity, respectively,


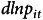
 and 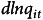 are the time rates of change of *p* and *q*, and 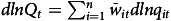 is the Divisia volume index of the aggregate quantity demanded. 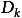 represents a seasonal dummy variable included to capture monthly effects, with twelve dummies accounting for seasonality in the thirteen statistical months per year. The parameter satisfies the constraint 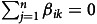 . 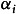 is the constant term of the *i* ^th^ demand equation satisfying the adding up restriction 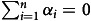 . The use of the constant term in the demand equations is to take into account any trend-like changes in tastes, etc. The parameter 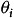 is the marginal share which satisfies 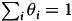 . This marginal share 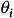 answers the question, ‘‘if income increases by one dollar, how much of this increase will be allocated to commodity i?’’ The 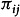 are the price coefficients in (1), which satisfies the adding-up restrictions 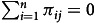 .

These price coefficients also satisfy the following constraints:
(2)

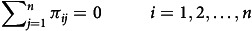




The above equation (2) reflects the homogeneity property of the demand system, which postulates that an equi-proportionate change in all prices does not affect the demand for any good under the condition that real income is constant.

The price coefficients are symmetric, that is:
(3)

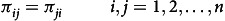




This means that an increase in the price of any good *j* will cause an increase in the compensated quantity demanded of *i* equal to the increase in the compensated quantity demanded of *j* caused by an increase in the price of *i*. Also, the Slutsky matrix 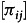 is symmetric and negative semi-definite with rank (*n*-1).

The term 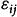 is the disturbance term of the *i* ^th^ equation. It is assumed that the disturbance terms, *ε*_*it*_ *, i =*1,…,*n*, are serially independent and normally distributed with zero means and with a contemporaneous covariance matrix. The income (total expenditure) elasticity implied by the demand system in equation (1) is given by
(4)

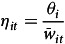




The compensated price elasticities associated with equation (1) are given by
(5)

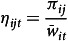




The uncompensated price elasticities are given by:
(6)

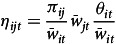




In equations (4), (5) and (6), 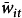 represents the arithmetic average of the budget shares in period *t* and *t* − 1 as aforementioned. As previously defined in the Rotterdam model specification, this term reflects the average share of expenditure allocated to the specific fish category under analysis. Although elasticity estimates are useful for measuring how consumer demand shifts in response to income and price changes, it is also important to understand the level of contribution of income and prices to consumption changes. Following the previous studies in the literature^(19,21,22)^, we used the estimation results for demand elasticities to decompose the growth in fish demand in terms of autonomous trend, effects of income, own-price and cross-price and seasonal effects.

Dividing both sides of the demand system in equation (1) by the budget share 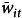 gives
(7)

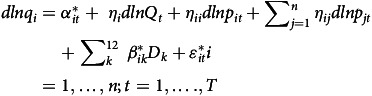




where 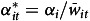 is the autonomous trend in consumption of item *i,* which measures the proportionate change in consumption of food item *i* in year *t* in the absence of changes in prices and income. The constant terms in differential demand systems represent trends, and the coefficients of seasonal dummies represent seasonal deviations from these trends^(30)^. Therefore, 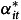 is generally interpreted as a trend effect due to the effect of changes in tastes and preferences. The coefficients 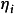 and 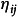 are expenditure and price elasticities. Therefore, growth in consumption of item *i* 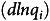 in each year can be decomposed into the following six components: (1) autonomous trend component ( 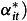 , (2) income component 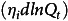 , (3) Own-price component 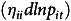 , (4) cross-price component 
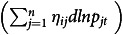
 , (5) seasonal component ( 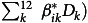 and (6) residual component 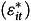 .

### Estimation approach and separability assumptions

Before estimating demand equations, we examined the stationarity of all variables in the demand systems to prevent spurious results. While first-differencing the data generally mitigates non-stationarity, we conducted the Augmented Dickey-Fuller unit root test^(31)^ as a precautionary measure given the extended time period covered in our analysis, which may include underlying trends. The test confirmed that all variables used in the demand systems were stationary*. We estimated separate demand systems for each of the above-mentioned seven household categories. This allowed us to capture the unique consumption patterns and preferences of different household types, leading to the calculation of distinct elasticities for each group. We subsequently assessed the homogeneity and symmetry of the demand theory hypotheses to ascertain their compatibility with the data. We used the sample size-corrected statistic^(32,33)^ (Appendix 1A) to test homogeneity and Slutsky symmetry. This statistical measure has been widely applied in empirical studies, as evidenced by various works in the literature^(34–36)^. According to the test results (Appendix 1B), we can infer that all the household groups, homogeneity and Slutsky symmetry at the 5 % significance level are consistent with the data. Therefore, the homogeneity and symmetry-restricted version of the Rotterdam demand equation (1) was estimated using the seemingly unrelated regression for each household group.

To clarify our assumptions regarding separability, we applied a multi-stage budgeting approach. This approach allows us to treat fish products as a distinct, separable category within the broader food expenditure group. We further decomposed demand into various types of fish products within this category, such as canned, fresh and frozen fish. The separability assumption implies that consumers first decide on an allocation of their budget to food and, within that, specifically to fish. The expenditure on fish is then allocated across different types of fish products without assuming additional separability between individual fish subgroups. This ensures that our demand elasticities are conditional on the initial budgetary decision to allocate expenditure to fish, providing a more precise understanding of substitution effects within the fish category.*

## Results

### Fish consumption patterns

**Figure 2.**
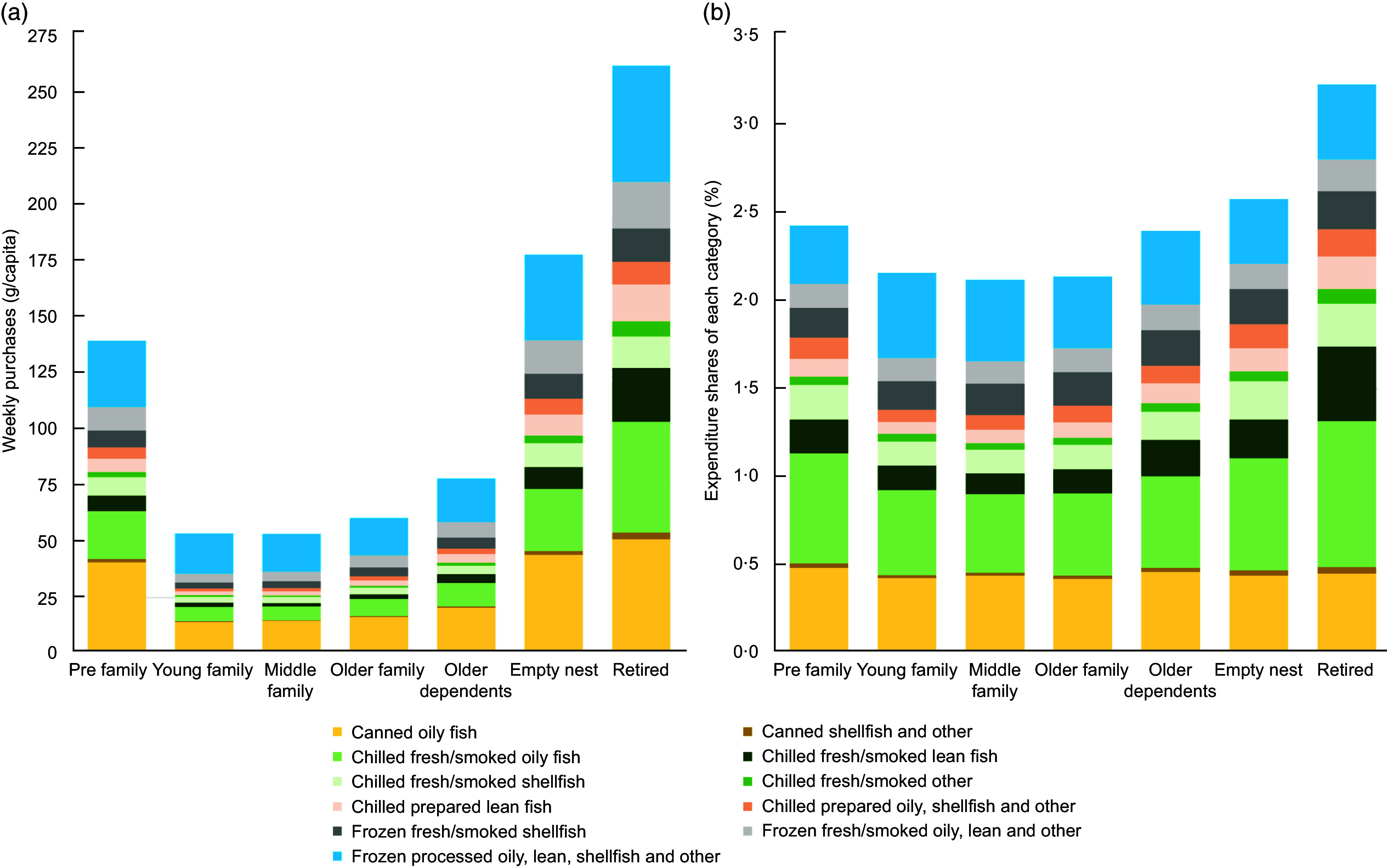
Analysis of weekly fish purchases (a) and expenditure shares (b) for each of the eleven fish subgroups.

Analysis of weekly fish purchasing behaviours highlighted distinct patterns among British consumers, notably with the highest mean values observed in the empty nest (178 g/capita) and retired groups (262 g/capita) (Figure 2(a)). Moreover, those in the empty nest and retired groups purchased more chilled fresh/smoked oily fish, indicating a desire for higher-quality, less processed fish options. Conversely, young and middle family categories purchased lower quantities of fish (54 g/capita), indicative of a much lower fish consumption. Among families with children, canned fish and frozen processed fish products were more popular than other fish groups. However, the popularity of canned oily fish, such as tuna or mackerel, was apparent across various household groups.

Retired households allocated the highest percentage of their grocery expenditure to fish purchases (3·23 %). Families with children consistently allocated a lower percentage of their total grocery expenditure to fish purchases, ranging from 2·12 % to 2·16 %. In contrast, households without children tended to allocate a slightly higher percentage on fish purchases, ranging from 2·40 % to 3·23 % (Figure 2(b)). Expenditure shares for canned oily fish and frozen processed fish items ranked second only to chilled fresh/smoked oily fish among the eleven fish categories.

### Demand elasticities

All own-price elasticities, e.g. the percentage change in the quantity demanded in response to a 1 % increase in price, for the eleven fish categories in the seven household groups, were negative. Most of these own-price elasticities were statistically significant at the 5 % level (Table 1). The demand for the chilled fresh/smoked oily was the most price-responsive, whereas demand for frozen fresh/smoked oily, lean and other fish was least responsive to price. However, most own-price elasticities were less than one in absolute values, indicating that the demand for fish products was inelastic in response to price changes. The magnitude of cross-price elasticities varied considerably across household groups but not in any systematic pattern (Appendix 2).

**Table 1.** Average uncompensated own-price elasticities, 2013–2021

	Pre family	Young family	Middle family	Older family	Older dependents	Empty nest	Retired
Canned oily fish	−0·526	−0·998	−0·533	−0·501	−0·532	−0·854	−0·499
	(0·1902)	(0·1819)	(0·2220)	(0·1585)	(0·1625)	(0·2113)	(0·1659)
Canned shellfish and other	−0·451	−0·457	−0·603	−0·261	−0·297	−0·327	−0·322
	(0·1434)	(0·1864)	(0·1643)	(0·1508)	(0·1789)	(0·1319)	(0·1417)
Chilled fresh/smoked oily fish	−0·994	−1·050	−0·906	−1·064	−0·759	−1·029	−1·109
	(0·0392)	(0·0437)	(0·361)	(0·0393)	(0·0547)	(0·0540)	(0·0327)
Chilled fresh/smoked lean fish	−0·985	−1·018	−0·205	−0·679	−0·608	−0·795	−0·667
	(0·2054)	(0·2865)	(0·102)	(0·2474)	(0·2734)	(0·1869)	(0·2738)
Chilled fresh/smoked shellfish	−0·343	−0·4833	−0·368	−0·066	−0·044	−0·369	−0·108
	(0·1170)	(0·1761)	(0·1166)	(0·0125)	(0·0017)	(0·1503)	(0·1507)
Chilled fresh/smoked other	−0·599	−0·299	−0·101	−0·523	−0·488	−0·076	−0·235
	(0·2292)	(0·1261)	(0·0046)	(0·2929)	(0·2105)	(0·0018)	(0·1933)
Chilled prepared lean fish	−0·202	−0·186	−0·184	−0·406	−0·107	−0·157	−0·042
	(0·2021)	(0·1018)	(0·011)	(0·1973)	(0·0214)	(0·0130)	(0·0030)
Chilled prepared oily, shellfish and other	−0·337	−0·416	−0·225	−0·495	−0·093	−0·279	−0·278
	(0·1507)	(0·1624)	(0·1700)	(0·1568)	(0·0028)	(0·0062)	(0·1632)
Frozen fresh/smoked shellfish	−0·574	−0·490	−0·622	−0·220	−0·406	−0·348	−0·397
	(0·1290)	(0·1273)	(0·1134)	(0·1178)	(0·1172)	(0·0983)	(0·1273)
Frozen fresh/smoked oily, lean and other	−0·231	−0·260	−0·185	−0·216	−0·052	−0·283	−0·194
	(0·0679)	(0·0682)	(0·0522)	(0·0586)	(0·0264)	(0·0528)	(0·0584)
Frozen processed oily, lean, shellfish and other	−0·276	−0·653	−0·481	−0·123	−0·099	−0·286	−0·231
	(0·1720)	(0·1257)	(0·1508)	(0·0028)	(0·0016)	(0·1544)	(0·1826)

Note: Standard errors are in parentheses.

All expenditure elasticities, e.g. the percentage change in quantity demanded of fish groups in response to a 1 % change in total household grocery expenditure, for all household groups, were positive and significant, except for that of canned shellfish and other and chilled prepared oily, shellfish and other in young families, which were positive but non-significant (Table 2). This implies the appeal of, and affordability for, fish for those in higher-income brackets. The varied expenditure elasticities for different product categories within family groups emphasise the varied nature of consumer choices. In all household groups, chilled prepared oily and shellfish and other products display relatively inelastic responses to income changes, indicating a consistent demand regardless of income fluctuations.

**Table 2.** Average expenditure elasticities, 2013–2021

	Pre family	Young family	Middle family	Older family	Older dependents	Empty nest	Retired
Canned oily fish	0·985	1·101	1·285	0·968	1·427	1·316	1·377
	(0·1354)	(0·1347)	(0·1237)	(0·1124)	(0·1293)	(0·1275)	(0·0994)
Canned shellfish and other	1·621	0·525	1·824	0·953	1·194	1·787	0·921
	(0·4069)	(0·5553)	(0·3748)	(0·3552)	(0·4071)	(0·3346)	(0·2298)
Chilled fresh/smoked oily fish	1·065	1·187	0·878	1·332	0·930	1·041	1·252
	(0·1216)	(0·1331)	(0·1360)	(0·1126)	(0·1412)	(0·0984)	(0·0822)
Chilled fresh/smoked lean fish	1·196	1·052	0·678	1·352	1·050	0·973	0·884
	(0·2065)	(0·3046)	(0·2464)	(0·2361)	(0·2155)	(0·1561)	(0·1276)
Chilled fresh/smoked shellfish	0·938	0·667	0·860	0·531	0·625	1·079	0·816
	(0·1568)	(0·2191)	(0·1608)	(0·1975)	(0·1717)	(0·1391)	(0·1233)
Chilled fresh/smoked other	0·743	1·025	1·341	1·055	0·730	1·246	1·150
	(0·3406)	(0·3935)	(0·4455)	(0·3796)	(0·2895)	(0·2999)	(0·1889)
Chilled prepared lean fish	0·673	0·795	1·026	1·220	0·813	0·407	0·573
	(0·2113)	(0·3170)	(0·2670)	(0·2354)	(0·2321)	(0·1813)	(0·1407)
Chilled prepared oily, shellfish and other	0·739	0·074	0·428	0·800	0·863	0·597	0·743
	(0·2371)	(0·3187)	(0·2651)	(0·2153)	(0·2672)	(0·2333)	(0·1817)
Frozen fresh/smoked shellfish	1·070	1·016	1·178	0·753	1·007	0·952	0·813
	(0·1554)	(0·1702)	(0·1406)	(0·1406)	(0·1487)	(0·1257)	(0·1221)
Frozen fresh/smoked oily, lean and other	1·135	1·139	1·372	0·830	1·010	1·165	0·914
	(0·1631)	(0·1744)	(0·1419)	(0·1511)	(0·1426)	(0·1400)	(0·1182)
Frozen processed oily, lean, shellfish and other	1·377	0·935	0·845	0·864	0·831	0·736	0·717
	(0·1325)	(0·0878)	(0·0866)	(0·1107)	(0·1184)	(0·1121)	(0·1080)

Note: Standard errors are in parentheses.

**Figure 3.**
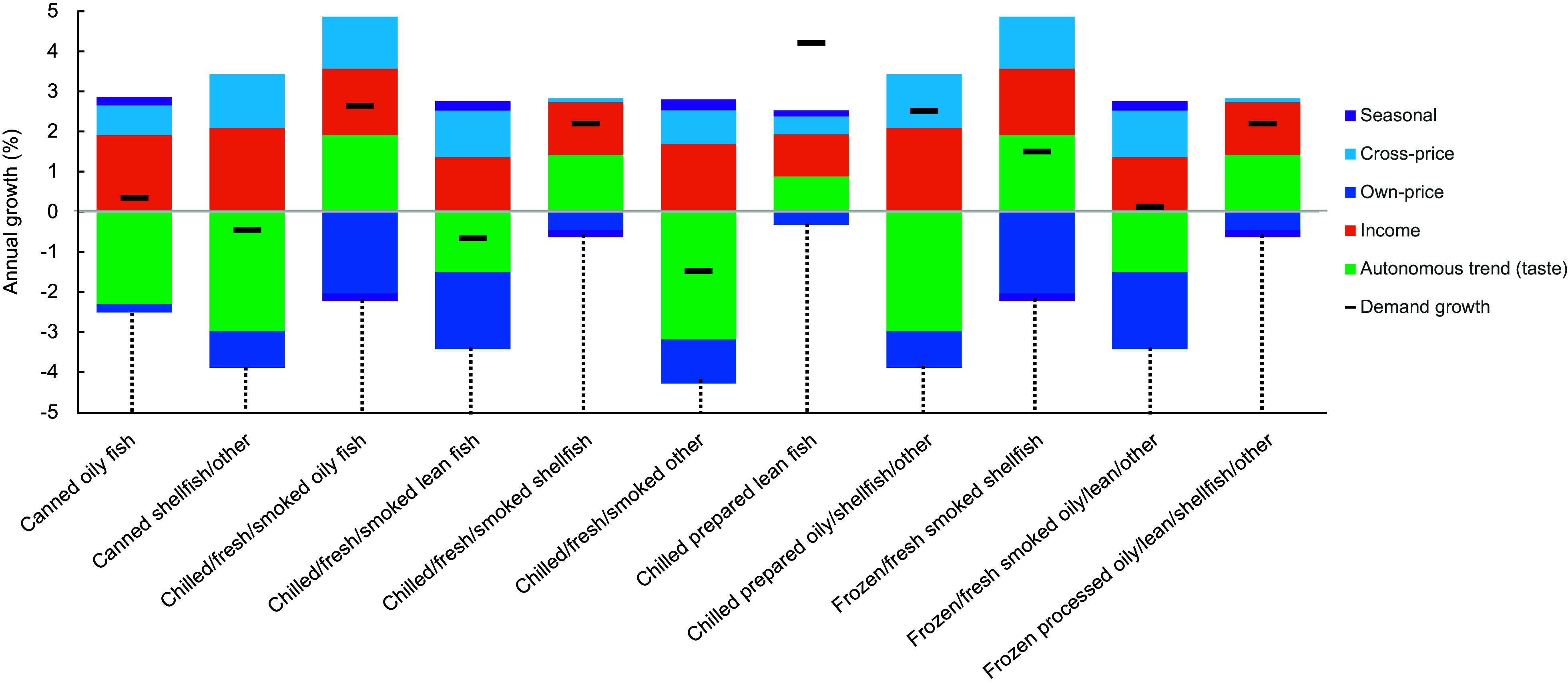
Average annual growth in demand and its components (seasonal, cross-price, own-price, income and autonomous trend) for the eleven fish subgroups from 2013 to 2021.

Figure 3 shows the average annual growth in demand and its components for eleven fish groups from 2013 to 2021. Among the eleven fish groups examined, distinct trends emerged. Notably, there was a positive growth trend observed in demand for chilled fresh/smoked oily fish, chilled fresh/smoked shellfish, chilled prepared lean fish and chilled prepared oily, shellfish and other varieties, with growth rates ranging from 2·19 % to 4·20 % annually. Conversely, certain categories experienced a decline in demand, such as canned shellfish and other (–0·46 %), chilled fresh/smoked lean fish (–0·67 %) and chilled fresh/smoked other (–1·48 %). Our analysis of expenditure shares, as illustrated in Figure 2(b), indicates that chilled fresh/smoked oily fish, canned oily fish and frozen processed fish products dominate spending across all demographic groups. However, the annual demand growth rate of canned oily fish remains below one percent in most household groups, suggesting a nuanced interplay of factors influencing consumer behaviour. Despite the current popularity and widespread consumption of canned oily fish, the sluggish growth rates may imply a potential stagnation or saturation in demand over time. For all fish groups, income and autonomous trends (changes in consumer taste) were the most important factors that affected changes in demand for fish. However, price played a comparatively dominant role in the consumption growth of the chilled fresh/smoked oily fish group, chilled fresh/smoked lean fish and frozen fresh fish products (Figure 3). The autonomous trend effects indicate that changes in consumer preferences have reoriented demand away from canned oily fish, canned shellfish and other group, chilled fresh/smoked lean fish, chilled fresh/smoked other fish, chilled prepared oily, shellfish and other, frozen fresh/smoked oily lean and other group, toward chilled fresh smoked oily fish, chilled fresh/smoked shellfish, chilled prepared lean fish and frozen fresh/smoked shellfish, frozen processed oily, lean, shellfish and other.

## Discussion

This study analysed the demand for fish for consumption at home across different household groups in Great Britain between 2013 and 2021. The analysis reveals several consumption behaviours. Notably, empty nesters and retirees purchase more fish compared with younger families. Additionally, this group also prefers higher quality, less processed options like chilled fresh/smoked oily fish. This finding is consistent with previous research showing age-related preferences for fish consumption with older people preferring fresh fish over processed varieties^(37–39)^. Families with children consistently allocate a lower share of their grocery spending on whole fish compared with households without children. These results suggest a link between age, household composition and seafood consumption habits. This aligns with prior studies indicating that older individuals tend to consume higher quantities of seafood^(40–42)^. Moreover, our observations complement existing research highlighting the health considerations driving seafood consumption among older demographics. With advancing age comes an increased risk of health issues such as cognitive decline and CVD, prompting dietary choices that prioritise nutrient-rich options like seafood^(41,43,44)^. In addition, we found that households with children who purchase fish preferred ready-to-use and convenient options, such as canned oily fish and frozen processed fish products. These findings are aligned with previous research indicating that changes in the perceived value of women’s time positively influence demand for processed fish and seafood products^(37)^. Canned oily fish, and frozen processed fish, rank second in expenditure shares among the eleven fish categories, following chilled fresh/smoked oily fish. This is also consistent with previous research showing a preference for ready-to-cook seafood products over whole or round-cut fish in European countries such as France, Germany, Italy and the UK^(45)^.

Our findings confirm that most fish categories are own-price inelastic, meaning that demand for these categories shows little response to price changes, a pattern also observed in previous studies^(14,15,46)^. At the same time, our study found positive and significant expenditure elasticities across household groups, indicating a consistent increase in demand in response to increases in total household grocery expenditure (Table 2). However, the magnitudes and patterns differ from those found by Burton & Young^(46)^, who found a unit expenditure elasticity (i.e. when income increases, people do not necessarily spend proportionally more on the same items). Notably, our analysis emphasises the appeal and affordability of certain fish products in higher-income brackets, such as canned shellfish and other for pre-family, middle family and empty nests and chilled fresh/smoked oily fish for young family, older family and retired groups, which corresponds to the observation by Burton^(13)^ about the expenditure elasticity of specific fish types, including white fish and flatfish despite the temporal gap of over three decades. Differences in applied methodologies, sample scope and product aggregation highlight the importance of the need for a nuanced interpretation of results when comparing studies based on economic data.

In all fish categories, income and the autonomous trend (shifts in consumer preferences) were key factors influencing changes in fish demand. This finding is consistent with previous behavioural studies on fish consumption, indicating that taste preferences towards fish and seafood play a pivotal role in shaping fish consumption behaviour^(47,48)^. Additionally, studies exploring the influence of socio-demographic factors on seafood consumption further support our findings, highlighting that income is a significant determinant of seafood consumption patterns^(40–42)^. Thus, contrary to the belief that low fish consumption is primarily due to affordability^(42,49)^, our findings, based on recent data and employing a demand system approach, suggest that the demand for most fish products across household groups is price inelastic. This indicates that while price does play a role, growth in demand is more significantly driven by household income and changes in consumer tastes.

With price having a limited effect on the demand for most fish products, increasing fish consumption, particularly among lower-income groups who do not usually consume much fish, may require more intervention than simply making fish more affordable, for example, through income support programmes such as cash transfers or food vouchers for lower-income households. Given that families with children consistently devote a smaller portion of their grocery budget to purchasing fish, which is in contrast to families without children, policymakers might consider targeted support or promotions for whole fish products for families with children. Such initiatives may include educational programmes focused on the nutritional benefits of whole fish, in-store tastings and special price promotions aimed at incentivising the trial and purchase of whole fish products at the point of sale^(48,50)^. To double fish consumption in households in Great Britain in order to meet dietary recommendations, policies should prioritise aligning offerings with consumer preferences^(51)^.

Strengths of this study include the use of the Kantar Worldpanel dataset, which holds data on fish purchases from well over twelve thousand households in Great Britain. The data we used for our analysis were collected over an extensive period of 9 years, providing longer-term information on the price and quantities purchased and the type of promotion used. One limitation of this study is that we performed demand analysis within the fish food group, and therefore, this study does not consider how price changes in other food groups may have affected demand for fish. Additionally, to simplify the analysis and ensure robust estimates, we aggregated fish subgroups, which reduced noise caused by infrequent consumption in certain subcategories. However, this approach may obscure zero consumption levels at more granular subgroup levels, representing a trade-off inherent in using aggregate data. Finally, the classification of the seven household groups follows Kantar’s categorisation of data, and therefore, we were not able to fully capture the diversity of consumers within each of these household groups.

In conclusion, we show that the demand for most fish products across household groups was less dependent on price and more dependent on income and change in taste. Thus, increasing the demand for fish and increasing fish consumption, especially in groups for which the potential health benefits matter most, such as those in lower-income groups who do not usually consume much fish, may require more than simply making fish more affordable. However, addressing the need for increased fish demand must be accompanied by a parallel focus on sustainable expansion of fish supplies, particularly targeting fish species with a high *n*-3 fatty acid, micronutrient and vitamin content, which are recognised for their health benefits. This approach ensures a balanced alignment between fish supply expansion and consumer preferences, particularly those demonstrating elasticity to income and price changes. Moreover, it mitigates the risk of upward pressure on fish prices, thereby preventing unintended consequences. The success of the above policy suggestions relies on collaborative efforts between government entities, the fish and seafood industry, retailers and consumer advocacy groups. By working together, these stakeholders can ensure a holistic and effective approach to sustainable fish market growth, thereby safeguarding public health and promoting access to nutritious seafood options for all consumers.

## Footnotes

* To save the space, results of unit root test are not included in the manuscript but are available upon the request.

## Appendices

### Appendix 1A

The calculation of the test statistic follows the approach outlined in Court (1968) and Deaton (1974);

where Ω^*R*^ and Ω^*U*^ denote the estimated residual covariance matrices with and without restrictions imposed, respectively, *T* is the number of observations, *n* is the number of equations in the system, *k* is the number of estimated parameters in each equation and *q* is the number of restrictions. The test statistic *F* follows an approximate distribution as *F[q, (n-1)(T–k)]*.

### Appendix 1B Testing demand homogeneity and Slutsky symmetry

**Table:**

	Homogeneity			Symmetry		
Household group	Test statistic	F _Critical_	Conclusion	Test statistic	F _Critical_	Conclusion
Pre family	2.15*	1.99	Accept	1.05	1.59	Accept
Young family	1.32	1.71	Accept	1.63*	1.59	Accept
Middle family	1.36	1.71	Accept	0.84	1.59	Accept
Older family	0.76	1.71	Accept	1.63*	1.59	Accept
Older dependents	1.47	1.71	Accept	1.68*	1.59	Accept
Empty nest	1.29	1.71	Accept	1.02	1.59	Accept
Retired	0.34	1.71	Accept	1.55	1.59	Accept

* denotes critical value and conclusion at *α* = 0·01.

### Appendix 2 Uncompensated price elasticities

**Table:**

	Canned oily fish	Canned shellfish and other	Chilled fresh/smoked oily fish	Chilled fresh/smoked lean fish	Chilled fresh/smoked shellfish	Chilled fresh/smoked other	Chilled prepared lean fish	Chilled prepared oily, shellfish and other	Frozen fresh/smoked shellfish	Frozen fresh/smoked oily, lean and other	Frozen processed oily, lean, shellfish and other
Pre family											
Canned oily fish	**−0·526**	−1·246	−0·028	0·259	−0·027	–0·0888	−0·986	0·509	−0·143	−0·298	−0·355
	**(0·1902)**	(0·5207)	(0·0560)	(0·2332)	(0·1694)	(0·4649)	(0·3019)	(0·2400)	(0·1892)	(0·1548)	(0·1565)
Canned fish, shellfish and other	−0·059	**−0·451**	0·009	0·009	−0·017	–0·2167	0·288	−0·020	−0·009	−0·053	0·010
	(0·0268)	**(0·1434)**	(0·0068)	(0·0443)	(0·0324)	(0·0937)	(0·0603)	(0·0454)	(0·0352)	(0·0283)	(0·0296)
Chilled fresh/smoked oily fish	−0·015	0·071	**−0·994**	0·086	0·057	–0·0592	−0·075	0·023	−0·018	0·007	−0·048
	(0·0437)	(0·1300)	**(0·0392)**	(0·0634)	(0·0502)	(0·1374)	(0·0680)	(0·0762)	(0·0496)	(0·0523)	(0·0340)
Chilled fresh/smoked lean fish	0·119	0·033	0·037	**−0·985**	−0·298	–0·5418	0·263	0·005	0·067	0·059	−0·137
	(0·0889)	(0·3300)	(0·0270)	**(0·2054)**	(0·1080)	(0·2911)	(0·1882)	(0·1575)	(0·1118)	(0·0994)	(0·0912)
Chilled fresh/smoked shellfish	−0·015	−0·189	0·008	−0·329	**−0·343**	–0·6442	0·439	−0·024	−0·098	−0·169	−0·092
	(0·0677)	(0·2530)	(0·0234)	(0·1133)	**(0·1170)**	(0·2222)	(0·1423)	(0·1222)	(0·0866)	(0·0766)	(0·0714)
Chilled fresh/smoked other	−0·013	−0·417	−0·011	−0·143	−0·158	**−0·599**	−0·257	0·131	0·021	0·039	0·024
	(0·0454)	(0·1734)	(0·0105)	(0·0739)	(0·0533)	**(0·2292)**	(0·1050)	(0·0742)	(0·0622)	(0·0463)	(0·0535)
Chilled prepared lean fish	−0·216	1·083	−0·028	0·116	0·211	–0·5472	**−0·202**	−0·382	−0·075	0·101	−0·055
	(0·0606)	(0·2334)	(0·0150)	(0·0980)	(0·0710)	(0·2215)	**(0·2021)**	(0·0987)	(0·0875)	(0·0624)	(0·0719)
Chilled prepared oily shellfish and other	0·115	−0·139	−0·012	−0·020	−0·024	0·3342	−0·458	**−0·337**	−0·108	−0·168	−0·136
	(0·0574)	(0·2146)	(0·0192)	(0·0995)	(0·0734)	(0·1891)	(0·1198)	**(0·1507)**	(0·0744)	(0·0678)	(0·0570)
Frozen fresh/smoked shellfish	−0·044	−0·098	−0·004	0·051	−0·074	0·0973	−0·099	−0·127	**−0·574**	−0·454	0·126
	(0·0630)	(0·2287)	(0·0190)	(0·0980)	(0·0721)	(0·2205)	(0·1462)	(0·1033)	**(0·1290)**	(0·0768)	(0·0716)
Frozen fresh/smoked oily, lean and other	−0·075	−0·306	0·005	0·039	−0·105	0·1332	0·163	−0·167	−0·363	**−0·231**	0·025
	(0·0420)	(0·1520)	(0·0166)	(0·0715)	(0·0524)	(0·1327)	(0·0862)	(0·1621)	(0·0623)	**(0·0679)**	(0·0414)
Frozen processed oily lean	−0·254	0·038	−0·046	−0·279	−0·159	0·1906	−0·151	−0·351	0·229	0·032	**−0·276**
shellfish and other	(0·1068)	(0·3877)	(0·0310)	(0·1617)	(0·1209)	(0·3760)	(0·2423)	(0·1575)	(0·1449)	(0·1032)	**(0·1720)**
Young family											
Canned oily fish	**−0·998**	−0·946	0·030	−0·522	0·296	–0·8975	0·097	0·180	0·331	−0·185	0·047
	**(0·1819)**	(0·6761)	(0·0587)	(0·2952)	(0·2255)	(0·5268)	(0·3786)	(0·2983)	(0·1783)	(0·1388)	(0·1097)
Canned fish, shellfish and other	−0·040	**−0·457**	0·001	0·059	0·003	–0·2267	0·004	0·000	0·004	−0·013	0·021
	(0·0247)	**(0·1864)**	(0·0071)	(0·0512)	(0·0420)	(0·1035)	(0·0734)	(0·0548)	(0·0338)	(0·0234)	(0·0196)
Chilled fresh/smoked oily fish	0·054	0·169	**−1·050**	0·077	0·000	–0·0787	0·048	0·149	−0·080	−0·004	−0·018
	(0·0436)	(0·1747)	**(0·0437)**	(0·0976)	(0·0700)	(0·1245)	(0·1025)	(0·1025)	(0·0547)	(0·0558)	(0·0285)
Chilled fresh/smoked lean fish	−0·172	0·537	0·013	**−1·018**	−0·267	0·4707	0·898	0·685	−0·172	−0·164	0·034
	(0·0932)	(0·4452)	(0·0347)	**(0·2865)**	(0·1587)	(0·3203)	(0·2529)	(0·2165)	(0·1149)	(0·0994)	(0·0668)
Chilled fresh/smoked shellfish	0·067	0·035	−0·033	−0·287	**−0·483**	0·1388	−0·182	0·032	0·028	−0·003	−0·090
	(0·0714)	(0·3642)	(0·0257)	(0·1582)	**(0·1761)**	(0·2662)	(0·2038)	(0·1677)	(0·0936)	(0·0753)	(0·0535)
Chilled fresh/smoked other	−0·093	−0·601	−0·010	0·148	0·052	**0·2989**	−0·223	−0·223	0·199	0·035	−0·073
	(0·0519)	(0·2790)	(0·0134)	(0·0991)	(0·0828)	**(0·1261)**	(0·1502)	(0·1060)	(0·0721)	(0·0468)	(0·0417)
Chilled prepared lean fish	0·006	0·025	−0·006	0·440	−0·088	–0·3608	**−0·186**	−0·217	−0·121	0·043	−0·126
	(0·0598)	(0·3154)	(0·0185)	(0·1255)	(0·1015)	(0·2395)	**(0·1018)**	(0·1336)	(0·0823)	(0·0580)	(0·0467)
Chilled prepared oily shellfish and other	−0·004	−0·013	−0·015	0·313	−0·003	–0·3858	−0·241	**−0·416**	−0·072	−0·083	−0·039
	(0·0473)	(0·2394)	(0·0181)	(0·1092)	(0·0847)	(0·1721)	(0·1353)	**(0·1624)**	(0·0617)	(0·0524)	(0·0351)
Frozen fresh/smoked shellfish	0·118	0·078	−0·039	−0·202	0·058	0·7308	−0·265	−0·095	**−0·490**	−0·538	−0·062
	(0·0654)	(0·3397)	(0·0252)	(0·1342)	(0·1091)	(0·2681)	(0·1918)	(0·1431)	**(0·1273)**	(0·0740)	(0·0518)
Frozen fresh/smoked oily, lean and other	−0·055	−0·071	−0·004	−0·152	0·026	0·1142	0·103	−0·093	0·127	**−0·260**	0·022
	(0·0421)	(0·1976)	(0·0211)	(0·0972)	(0·0734)	(0·1474)	(0·1133)	(0·1018)	(0·0613)	**(0·0682)**	(0·0301)
Frozen processed oily lean shellfish and other	0·016	0·720	−0·074	0·094	−0·260	–0·8291	−0·849	−0·076	−0·203	0·034	**−0·653**
	(0·1240)	(0·6004)	(0·0504)	(0·2404)	(0·1907)	(0·4706)	(0·3324)	(0·2507)	(0·1597)	(0·1147)	**(0·1257)**
Middle family											
Canned oily fish	**−0·533**	−0·825	0·061	−0·026	−0·096	−1·971	0·083	−0·750	−0·025	−0·251	−0·241
	**(0·2220)**	(0·6796)	(0·0559)	(0·3269)	(0·2071)	(0·6709)	(0·3771)	(0·3127)	(0·1824)	(0·1314)	(0·1071)
Canned fish, shellfish and other	−0·027	**−0·603**	0·008	−0·097	0·011	0·053	−0·063	−0·076	0·032	0·038	0·023
	(0·0253)	**(0·1643)**	(0·0049)	(0·0472)	(0·0299)	(0·1025)	(0·0574)	(0·0444)	(0·0292)	(0·0180)	(0·0208)
Chilled fresh/smoked oily fish	−0·024	0·017	**−0·906**	−0·066	−0·059	0·175	0·019	−0·041	0·018	0·037	−0·061
	(0·0335)	(0·1040)	**(0·361)**	(0·0671)	(0·0450)	(0·1220)	(0·0730)	(0·0713)	(0·0378)	(0·0377)	(0·0236)
Chilled fresh/smoked lean fish	−0·041	−0·743	−0·029	**−0·205**	0·008	0·157	−0·134	−0·245	−0·015	−0·052	−0·035
	(0·0850)	(0·3328)	(0·0227)	**(0·102)**	(0·1132)	(0·3358)	(0·1982)	(0·1680)	(0·0880)	(0·0715)	(0·0671)
Chilled fresh/smoked shellfish	0·085	0·029	−0·019	0·021	**−0·368**	−0·218	−0·232	0·036	−0·066	0·021	−0·053
	(0·0632)	(0·2481)	(0·0188)	(0·1332)	**(0·1166)**	(0·2526)	(0·1492)	(0·1272)	(0·0664)	(0·0544)	(0·0493)
Chilled fresh/smoked other	−0·154	0·102	0·021	0·057	−0·047	**0·101**	0·118	0·000	0·007	−0·070	0·037
	(0·0514)	(0·2121)	(0·0119)	(0·0986)	(0·0636)	**(0·0046)**	(0·1180)	(0·0967)	(0·0540)	(0·0399)	(0·0392)
Chilled prepared lean fish	0·005	−0·316	0·009	−0·074	−0·123	0·249	**−0·184**	0·160	0·044	−0·119	−0·113
	(0·0637)	(0·2627)	(0·0163)	(0·1285)	(0·0823)	(0·2607)	**(0·011)**	(0·1249)	(0·0685)	(0·0527)	(0·0492)
Chilled prepared oily shellfish and other	−0·181	−0·447	−0·026	−0·189	0·005	−0·036	0·156	**−0·225**	0·130	−0·015	0·048
	(0·0580)	(0·2262)	(0·0173)	(0·1211)	(0·0779)	(0·2376)	(0·1389)	**(0·1700)**	(0·0626)	(0·0531)	(0·0442)
Frozen fresh/smoked shellfish	−0·019	0·284	0·032	0·018	−0·059	0·020	0·113	0·329	**−0·622**	−0·536	−0·087
	(0·0719)	(0·3100)	(0·0232)	(0·1341)	(0·0860)	(0·2789)	(0·1604)	(0·1335)	**(0·1134)**	(0·0676)	(0·0559)
Frozen fresh/smoked oily, lean and other	−0·068	0·267	0·040	−0·015	0·051	−0·257	−0·178	0·034	−0·377	**−0·185**	−0·035
	(0·0377)	(0·1414)	(0·0168)	(0·0802)	(0·0518)	(0·1521)	(0·0911)	(0·0832)	(0·0492)	**(0·0522)**	(0·0286)
Frozen processed oily lean shellfish and other	−0·188	0·411	−0·070	−0·100	−0·183	0·385	−0·725	0·349	−0·302	−0·241	**−0·481**
	(0·1451)	(0·5897)	(0·0473)	(0·2728)	(0·1711)	(0·5410)	(0·3078)	(0·2527)	(0·1506)	(0·1071)	**(0·1508)**
Older family											
Canned oily fish	**−0·501**	0·412	−0·019	−0·086	0·094	−0·958	−0·207	0·170	−0·277	0·016	−0·300
	**(0·1585)**	(0·4978)	(0·0496)	(0·2554)	(0·1895)	(0·5078)	(0·2788)	(0·2352)	(0·1459)	(0·1158)	(0·1445)
Canned fish, shellfish and other	0·016	**−0·261**	−0·015	−0·003	−0·097	0·083	0·153	−0·071	−0·068	−0·033	0·024
	(0·0197)	**(0·1508)**	(0·0056)	(0·0411)	(0·0299)	(0·0993)	(0·0494)	(0·0399)	(0·0273)	(0·0165)	(0·0246)
Chilled fresh/smoked oily fish	0·058	−0·344	**−1·064**	−0·002	−0·038	0·395	0·104	0·020	−0·010	−0·020	−0·010
	(0·0391)	(0·1281)	**(0·0393)**	(0·0853)	(0·0680)	(0·1359)	(0·0829)	(0·0751)	(0·0486)	(0·0511)	(0·0394)
Chilled fresh/smoked lean fish	−0·003	0·003	0·001	**−0·679**	0·028	−0·836	−0·267	0·349	−0·129	0·031	−0·013
	(0·0812)	(0·3324)	(0·0298)	**(0·2474)**	(0·1337)	(0·3419)	(0·1925)	(0·1655)	(0·0978)	(0·0812)	(0·0916)
Chilled fresh/smoked shellfish	0·003	−0·835	−0·064	−0·025	**−0·066**	0·125	−0·343	0·110	−0·003	−0·071	−0·146
	(0·0631)	(0·2526)	(0·0259)	(0·1403)	**(0·0125)**	(0·2622)	(0·1511)	(0·1311)	(0·0774)	(0·0658)	(0·0711)
Chilled fresh/smoked other	−0·083	0·185	0·027	−0·235	0·042	**−0·523**	−0·103	−0·090	0·028	0·037	−0·010
	(0·0444)	(0·2193)	(0·0128)	(0·0935)	(0·0684)	**(0·2929)**	(0·1103)	(0·0894)	(0·0573)	(0·0386)	(0·0523)
Chilled prepared lean fish	−0·033	0·805	0·015	−0·178	−0·187	−0·235	**−0·406**	−0·193	0·147	−0·016	−0·017
	(0·0569)	(0·2559)	(0·0189)	(0·1235)	(0·0925)	(0·2585)	**(0·1973)**	(0·1192)	(0·0736)	(0·0536)	(0·0658)
Chilled prepared oily shellfish and other	0·031	−0·413	−0·020	0·222	0·088	−0·245	−0·231	**−0·495**	0·089	0·140	−0·233
	(0·0535)	(0·2297)	(0·0191)	(0·1184)	(0·0895)	(0·2333)	(0·1329)	**(0·1568)**	(0·0665)	(0·0524)	(0·0612)
Frozen fresh/smoked shellfish	−0·145	−0·786	−0·056	−0·234	0·016	0·115	0·278	0·172	**−0·220**	−0·537	0·021
	(0·0659)	(0·3127)	(0·0260)	(0·1397)	(0·1056)	(0·2971)	(0·1636)	(0·1328)	**(0·1178)**	(0·0739)	(0·0786)
Frozen fresh/smoked oily, lean and other	−0·004	−0·271	−0·038	−0·002	−0·049	0·121	−0·049	0·198	−0·375	**−0·216**	−0·056
	(0·0368)	(0·1352)	(0·0194)	(0·0827)	(0·0638)	(0·1439)	(0·0852)	(0·0745)	(0·0522)	**(0·0586)**	(0·0381)
Frozen processed oily lean shellfish and other	−0·309	0·552	−0·098	−0·131	−0·361	−0·143	−0·149	−0·970	0·065	−0·161	**−0·123**
	(0·1383)	(0·5935)	(0·0487)	(0·2756)	(0·2041)	(0·5716)	(0·3077)	(0·2568)	(0·1662)	(0·1155)	**(0·0028)**
Older dependents											
Canned oily fish	**−0·532**	−0·241	0·328	0·130	−0·073	–0·5694	0·065	0·703	0·443	0·266	−0·266
	**(0·1625)**	(0·4917)	(0·0438)	(0·2229)	(0·1677)	(0·3697)	(0·2748)	(0·2464)	(0·1504)	(0·1205)	(0·1362)
Canned fish, shellfish and other	−0·013	**−0·297**	0·016	−0·128	−0·005	–0·1335	−0·018	0·064	0·076	−0·027	0·057
	(0·0269)	**(0·1789)**	(0·0075)	(0·0509)	(0·0370)	(0·0991)	(0·0642)	(0·0529)	(0·0358)	(0·0249)	(0·0319)
Chilled fresh/smoked oily fish	0·368	0·317	**−0·759**	0·224	0·011	0·2093	0·106	0·165	0·218	0·151	0·148
	(0·0491)	(0·1537)	**(0·0547)**	(0·0817)	(0·0653)	(0·1105)	(0·0881)	(0·1011)	(0·0565)	(0·0541)	(0·0452)
Chilled fresh/smoked lean fish	0·059	−1·051	0·090	**−0·608**	0·121	0·1601	0·170	−0·236	−0·058	0·198	0·093
	(0·1004)	(0·4177)	(0·0327)	**(0·2734)**	(0·1459)	(0·3300)	(0·2331)	(0·2081)	(0·1276)	(0·1016)	(0·1151)
Chilled fresh/smoked shellfish	−0·025	−0·031	0·003	0·093	**−0·044**	–0·0096	0·191	0·318	−0·184	−0·084	−0·013
	(0·0581)	(0·2337)	(0·0202)	(0·1122)	**(0·0017)**	(0·1814)	(0·1290)	(0·1226)	(0·0713)	(0·0610)	(0·0649)
Chilled fresh/smoked other	−0·060	−0·257	0·020	0·038	−0·003	**−0·488**	0·164	−0·130	−0·082	−0·058	0·142
	(0·0391)	(0·1911)	(0·0104)	(0·0775)	(0·0554)	**(0·2105)**	(0·0963)	(0·0785)	(0·0529)	(0·0364)	(0·0488)
Chilled prepared lean fish	0·016	−0·083	0·024	0·094	0·138	0·3871	**−0·107**	−0·319	0·067	0·123	−0·188
	(0·0686)	(0·2919)	(0·0195)	(0·1292)	(0·0929)	(0·2272)	**(0·0214)**	(0·1351)	(0·0842)	(0·0634)	(0·0760)
Chilled prepared oily shellfish and other	0·151	0·250	0·032	−0·113	0·197	–0·2652	−0·275	**−0·093**	−0·080	−0·057	−0·054
	(0·0530)	(0·2074)	(0·0194)	(0·0994)	(0·0762)	(0·1597)	(0·1165)	**(0·0028)**	(0·0642)	(0·0545)	(0·0560)
Frozen fresh/smoked shellfish	0·194	0·604	0·085	−0·056	−0·232	–0·3375	0·117	−0·162	**−0·406**	−0·484	0·172
	(0·0657)	(0·2853)	(0·0220)	(0·1238)	(0·0899)	(0·2188)	(0·1476)	(0·1305)	**(0·1172)**	(0·0767)	(0·0729)
Frozen fresh/smoked oily, lean and other	0·084	−0·154	0·043	0·139	−0·076	–0·1752	0·157	−0·084	−0·351	**−0·052**	0·008
	(0·0382)	(0·1440)	(0·0153)	(0·0715)	(0·0558)	(0·1092)	(0·0806)	(0·0804)	(0·0556)	**(0·0264)**	(0·0396)
Frozen processed oily lean shellfish and other	−0·241	0·941	0·119	0·187	−0·033	1·222	−0·684	−0·226	0·356	0·023	**−0·099**
	(0·1237)	(0·5290)	(0·0365)	(0·2321)	(0·1703)	(0·4198)	(0·2767)	(0·2364)	(0·1515)	(0·1135)	**(0·0016)**
Empty nest											
Canned oily fish	**−0·854**	−1·711	0·047	−0·344	−0·086	−1·1574	0·283	0·414	0·177	0·118	−0·078
	**(0·2113)**	(0·4567)	(0·0503)	(0·2145)	(0·1827)	(0·4101)	(0·2582)	(0·2690)	(0·1464)	(0·1298)	(0·1487)
Canned fish, shellfish and other	−0·112	**−0·327**	0·018	0·049	−0·021	0·0761	0·018	−0·055	−0·025	0·025	0·036
	(0·0304)	**(0·1319)**	(0·0082)	(0·0410)	(0·0368)	(0·0839)	(0·0551)	(0·0532)	(0·0298)	(0·0246)	(0·0291)
Chilled fresh/smoked oily fish	0·000	0·207	**−1·029**	0·243	0·015	0·0573	−0·032	−0·022	−0·121	−0·055	−0·020
	(0·0654)	(0·1723)	**(0·0540)**	(0·0805)	(0·0709)	(0·1533)	(0·0923)	(0·1203)	(0·0637)	(0·0712)	(0·0588)
Chilled fresh/smoked lean fish	−0·200	0·284	0·077	**−0·795**	−0·045	0·0498	−0·031	0·116	0·086	−0·084	−0·067
	(0·1042)	(0·3005)	(0·0289)	**(0·1869)**	(0·1180)	(0·2690)	(0·1740)	(0·1694)	(0·0946)	(0·0833)	(0·0921)
Chilled fresh/smoked shellfish	−0·063	−0·210	0·008	−0·036	**−0·369**	–0·0925	0·240	−0·160	−0·037	−0·130	−0·217
	(0·0903)	(0·2736)	(0·0264)	(0·1200)	**(0·1503)**	(0·2446)	(0·1607)	(0·1534)	(0·0854)	(0·0737)	(0·0840)
Chilled fresh/smoked other	−0·150	0·131	0·010	0·019	−0·020	**–0·0760**	−0·207	0·172	−0·065	−0·009	0·060
	(0·0507)	(0·1565)	(0·0137)	(0·0685)	(0·0612)	**(0·0018)**	(0·0924)	(0·0872)	(0·0499)	(0·0416)	(0·0489)
Chilled prepared lean fish	0·038	0·008	−0·038	−0·047	0·109	–0·5178	**−0·157**	−0·381	0·012	0·019	−0·108
	(0·0734)	(0·2360)	(0·0196)	(0·1019)	(0·0923)	(0·2123)	**(0·0130)**	(0·1226)	(0·0803)	(0·0575)	(0·0747)
Chilled prepared oily shellfish and other	0·090	−0·310	−0·028	0·052	−0·124	0·3773	−0·387	**−0·279**	−0·141	−0·181	−0·029
	(0·0807)	(0·2415)	(0·0263)	(0·1046)	(0·0933)	(0·2122)	(0·1295)	**(0·0062)**	(0·0731)	(0·0693)	(0·0703)
Frozen fresh/smoked shellfish	0·052	−0·235	−0·045	0·078	−0·044	–0·2538	0·061	−0·182	**−0·348**	−0·556	−0·041
	(0·0641)	(0·1983)	(0·0214)	(0·0858)	(0·0761)	(0·1778)	(0·1247)	(0·1077)	**(0·0983)**	(0·0638)	(0·0634)
Frozen fresh/smoked oily, lean and other	0·030	0·083	−0·006	−0·044	−0·081	–0·0276	0·063	−0·161	−0·385	**−0·283**	0·013
	(0·0406)	(0·1174)	(0·0168)	(0·0539)	(0·0470)	(0·1067)	(0·0639)	(0·0731)	(0·0456)	**(0·0528)**	(0·0359)
Frozen processed oily lean shellfish and other	−0·147	0·294	−0·055	−0·147	−0·413	0·3183	−0·259	−0·060	−0·105	−0·028	**−0·286**
	(0·1200)	(0·3549)	(0·0379)	(0·1536)	(0·1374)	(0·3203)	(0·2131)	(0·1908)	(0·1171)	(0·0936)	**(0·1544)**
Retired											
Canned oily fish	**−0·499**	−0·156	0·043	−0·528	0·147	–0·0990	−0·357	0·289	0·255	0·124	−0·274
	**(0·1659)**	(0·3380)	(0·0289)	(0·1614)	(0·1532)	(0·2736)	(0·1861)	(0·2019)	(0·1422)	(0·1090)	(0·1322)
Canned fish, shellfish and other	−0·019	**−0·322**	−0·011	−0·032	−0·015	0·3101	0·047	−0·012	−0·025	−0·018	−0·039
	(0·0287)	**(0·1417)**	(0·0052)	(0·0384)	(0·0375)	(0·0788)	(0·0472)	(0·0454)	(0·0396)	(0·0241)	(0·0334)
Chilled fresh/smoked oily fish	0·047	−0·140	**−1·109**	−0·071	0·165	0·1008	0·037	0·133	−0·022	0·027	0·068
	(0·0389)	(0·0902)	**(0·0327)**	(0·0506)	(0·0481)	(0·0752)	(0·0553)	(0·0710)	(0·0477)	(0·0460)	(0·0425)
Chilled fresh/smoked lean fish	−0·558	−0·355	−0·084	**−0·667**	−0·483	0·0809	0·763	−0·412	0·033	0·320	0·383
	(0·1485)	(0·4171)	(0·0330)	**(0·2738)**	(0·1943)	(0·3367)	(0·2332)	(0·2464)	(0·1751)	(0·1354)	(0·1606)
Chilled fresh/smoked shellfish	0·037	−0·104	0·016	−0·283	**−0·108**	–0·5517	−0·124	0·054	0·126	−0·130	−0·154
	(0·0821)	(0·2375)	(0·0184)	(0·1132)	**(0·1507)**	(0·1934)	(0·1275)	(0·1391)	(0·0969)	(0·0745)	(0·0897)
Chilled fresh/smoked other	−0·025	0·690	0·008	0·023	−0·185	**−0·235**	−0·100	−0·154	−0·175	−0·093	0·011
	(0·0510)	(0·1731)	(0·0096)	(0·0680)	(0·0672)	**(0·1933)**	(0·0857)	(0·0820)	(0·0677)	(0·0438)	(0·0592)
Chilled prepared lean fish	−0·193	0·207	−0·031	0·318	−0·109	–0·2502	**−0·042**	0·006	0·011	−0·119	−0·333
	(0·0760)	(0·2273)	(0·0161)	(0·1034)	(0·0971)	(0·1881)	**(0·0030)**	(0·1228)	(0·0904)	(0·0657)	(0·0823)
Chilled prepared oily shellfish and other	0·068	−0·058	0·000	−0·156	0·031	–0·2971	0·013	**−0·278**	−0·123	−0·077	−0·041
	(0·0678)	(0·1807)	(0·0160)	(0·0899)	(0·0873)	(0·1484)	(0·1013)	**(0·1632)**	(0·0775)	(0·0635)	(0·0742)
Frozen fresh/smoked shellfish	0·084	−0·145	−0·035	0·012	0·113	–0·4664	0·029	−0·169	**−0·397**	−0·534	−0·024
	(0·0669)	(0·2213)	(0·0161)	(0·0896)	(0·0851)	(0·1723)	(0·1046)	(0·1087)	**(0·1273)**	(0·0714)	(0·0771)
Frozen fresh/smoked oily, lean and other	0·024	−0·083	−0·013	0·138	−0·091	–0·2098	−0·097	−0·081	−0·438	**−0·194**	−0·083
	(0·0422)	(0·1105)	(0·0130)	(0·0571)	(0·0539)	(0·0916)	(0·0626)	(0·0737)	(0·0591)	**(0·0584)**	(0·0443)
Frozen processed oily lean shellfish and other	−0·344	−0·455	−0·035	0·363	−0·281	–0·0021	−0·743	−0·117	−0·061	−0·221	**−0·231**
(0·1223)	(0·1223)	(0·3646)	(0·0303)	(0·1616)	(0·1549)	(0·2942)	(0·1869)	(0·2047)	(0·1511)	(0·1066)	**(0·1826)**

Note: Standard errors are in parentheses. Own-price elasticities are shown in bold for ease of identification.
